# New approaches to targeted drug therapy of intracranial tumors

**DOI:** 10.1038/s41420-025-02358-3

**Published:** 2025-03-20

**Authors:** Ozal Beylerli, Ilgiz Gareev, Elmar Musaev, Sergey Roumiantsev, Vladimir Chekhonin, Aamir Ahmad, Yuan Chao, Guang Yang

**Affiliations:** 1 Central Research Laboratory, Bashkir State Medical University, Ufa, Republic of Bashkortostan Russian Federation; 2 Sechenov First Moscow State Medical University, Moscow, Russian Federation; 3 Pirogov Russian National Research Medical University of the Ministry of Healthcare of Russian Federation, Moscow, Russian Federation; 4 Serbsky Federal Medical Research Centre of Psychiatry and Narcology of the Ministry of Healthcare of Russian Federation, Moscow, Russian Federation; 5 Endocrinology Research Center, Moscow, Russian Federation; 6 Translational Research Institute, Academic Health System, Hamad Medical Corporation, Doha, Qatar; 7 Department of Neurosurgery, The First Affiliated Hospital of Harbin Medical University, Harbin, 150001 Heilongjiang Province China; 8 Heilongjiang Province Neuroscience Institute, Harbin, China

**Keywords:** Molecular neuroscience, CNS cancer

## Abstract

Intracranial tumors encompass a heterogeneous group of neoplasms, including gliomas, meningiomas, pituitary adenomas, schwannomas, craniopharyngiomas, ependymomas, medulloblastomas, and primary central nervous system lymphomas. These tumors present significant challenges due to their diverse molecular characteristics, critical locations, and the unique obstacles posed by the blood-brain barrier (BBB) and blood-tumor barrier (BTB), which limit the efficacy of systemic therapies. Recent advances in molecular biology and genomics have enabled the identification of specific molecular pathways and targets, paving the way for innovative precision therapies. This review examines the current state of targeted therapies for intracranial tumors, including receptor tyrosine kinase (RTK) inhibitors, PI3K/AKT/mTOR inhibitors, RAF/MEK/ERK pathway inhibitors, IDH mutation inhibitors, immune checkpoint inhibitors, and CAR-T cell therapies. Emphasis is placed on the role of the BBB and BTB in modulating drug delivery and therapeutic outcomes. Strategies to overcome these barriers, such as focused ultrasound, nanoparticle-based delivery systems, and convection-enhanced delivery, are also explored. Furthermore, the manuscript reviews clinical trial data, highlighting successes and limitations across different tumor types. It delves into emerging therapeutic approaches, including combination of regimens and personalized treatments based on molecular profiling. By synthesizing the latest research, this article aims to provide a comprehensive understanding of the advancements and ongoing challenges in the targeted treatment of intracranial tumors. The findings underscore the necessity for innovative delivery systems and more extensive clinical trials to optimize therapeutic strategies. This review aspires to inform future research and clinical practices, aiming to improve patient outcomes and quality of life in the management of these complex and life-threatening conditions.

## Facts

- Blood–Brain Barrier and Blood–Tumor Barrier. The BBB and BTB significantly impede the effective delivery of systemic therapies to intracranial tumors. Innovative strategies, such as focused ultrasound, nanoparticles, and convection-enhanced delivery, show potential to overcome these barriers but require further clinical validation.
- Targeted pathway inhibitors. Molecular pathway inhibitors, such as those targeting RTKs, PI3K/AKT/mTOR, and RAF/MEK/ERK, offer promise in treating specific tumor subtypes. However, clinical outcomes remain inconsistent due to adaptive resistance and insufficient tumor site drug delivery.
- BRAF/MEK inhibitors for BRAF-V600E mutations. The combination of BRAF and MEK inhibitors has demonstrated high efficacy in managing papillary craniopharyngiomas and other tumors with BRAF-V600E mutations, highlighting the potential of precision medicine.
- Immunotherapy in brain tumors. Immune checkpoint inhibitors and CAR-T therapies hold promise but face challenges due to immunosuppressive tumor microenvironments, low mutational burdens in certain tumors, and the restrictive nature of the BBB.
- Personalized and combination approaches. The integration of targeted therapies with conventional treatments (e.g., chemotherapy, radiotherapy) or immunotherapies may address tumor heterogeneity and resistance, underscoring the need for patient-specific treatment regimens.

## Open Questions

- How can therapeutic delivery systems be further optimized to bypass or modulate the BBB and BTB effectively across all intracranial tumor types?
- What strategies can be developed to overcome resistance mechanisms associated with molecular pathway inhibitors in high-grade gliomas and other aggressive intracranial tumors?
- What are the synergistic effects of combining targeted therapies with immunotherapies or traditional treatments, and how can these combinations be tailored to individual tumor profiles?
- How can molecular profiling techniques be refined to enhance the precision of patient-specific therapy selection, particularly for heterogeneous tumors?
- What are the long-term safety profiles and clinical outcomes of emerging targeted therapies, and how can clinical trials better measure meaningful endpoints like quality of life and overall survival?

## Introduction

Intracranial tumors pose a significant and growing health challenge, particularly in regions such as Russia and China, where the incidence and mortality rates surpass global averages. These tumors can be broadly categorized into primary brain tumors, which originate from tissues within the brain such as the neuroepithelium, meninges, cranial nerves, and brain parenchyma, and secondary (metastatic) tumors, which spread to the brain from other organs or tissues [1]. Despite advances in surgical techniques, radiotherapy, and the development of new chemotherapy drugs, the prognosis for patients with intracranial tumors, particularly those located in critical brain regions or those with high malignancy, remains poor. Tumors in these areas present unique challenges due to their proximity to vital brain functions, which complicates surgical intervention and limits the effectiveness of traditional therapies. As a result, there has been a growing interest in targeted drug therapy, which offers the promise of selectively targeting tumor cells while minimizing damage to surrounding healthy tissues.

**Fig. 1:**
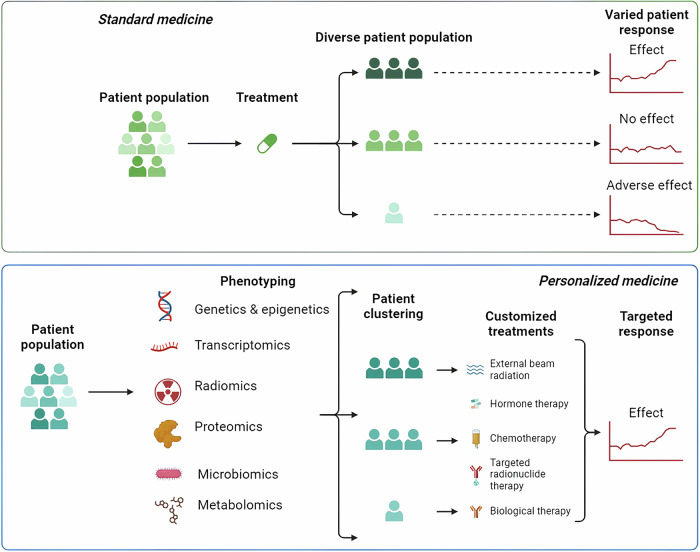
The potential of personalized medicine over standard treatment methods.

Intracranial tumors pose a significant challenge to human health due to their complex and often critical locations within the brain, leading to significant morbidity and mortality. Unlike many other types of tumors, intracranial neoplasms present unique treatment difficulties that go beyond the molecular targeting of cancer cells. The presence of the blood-brain barrier (BBB) and the blood-tumor barrier (BTB) severely limits the delivery of therapeutic agents, making it difficult to achieve effective drug concentrations at the tumor site [2]. This critical aspect differentiates targeted drug therapies for intracranial tumors from those for tumors located in other parts of the body. Advancements in molecular biology and genomics have made it possible to identify specific molecular targets, allowing for more precise treatments that selectively attack tumor cells. However, while targeted therapies are designed to home in on cancer cells regardless of their location, the context of intracranial tumors presents additional challenges due to their distinct physiological environment. This review will examine the landscape of targeted drug therapies specifically for intracranial tumors, discussing the mechanisms these therapies employ to overcome the obstacles posed by the BBB and BTB [2, 3]. Additionally, it will explore emerging techniques that aim to enhance drug delivery and efficacy, with the goal of improving clinical outcomes for patients suffering from these aggressive and complex conditions (Fig. 1).

**Fig. 2:**
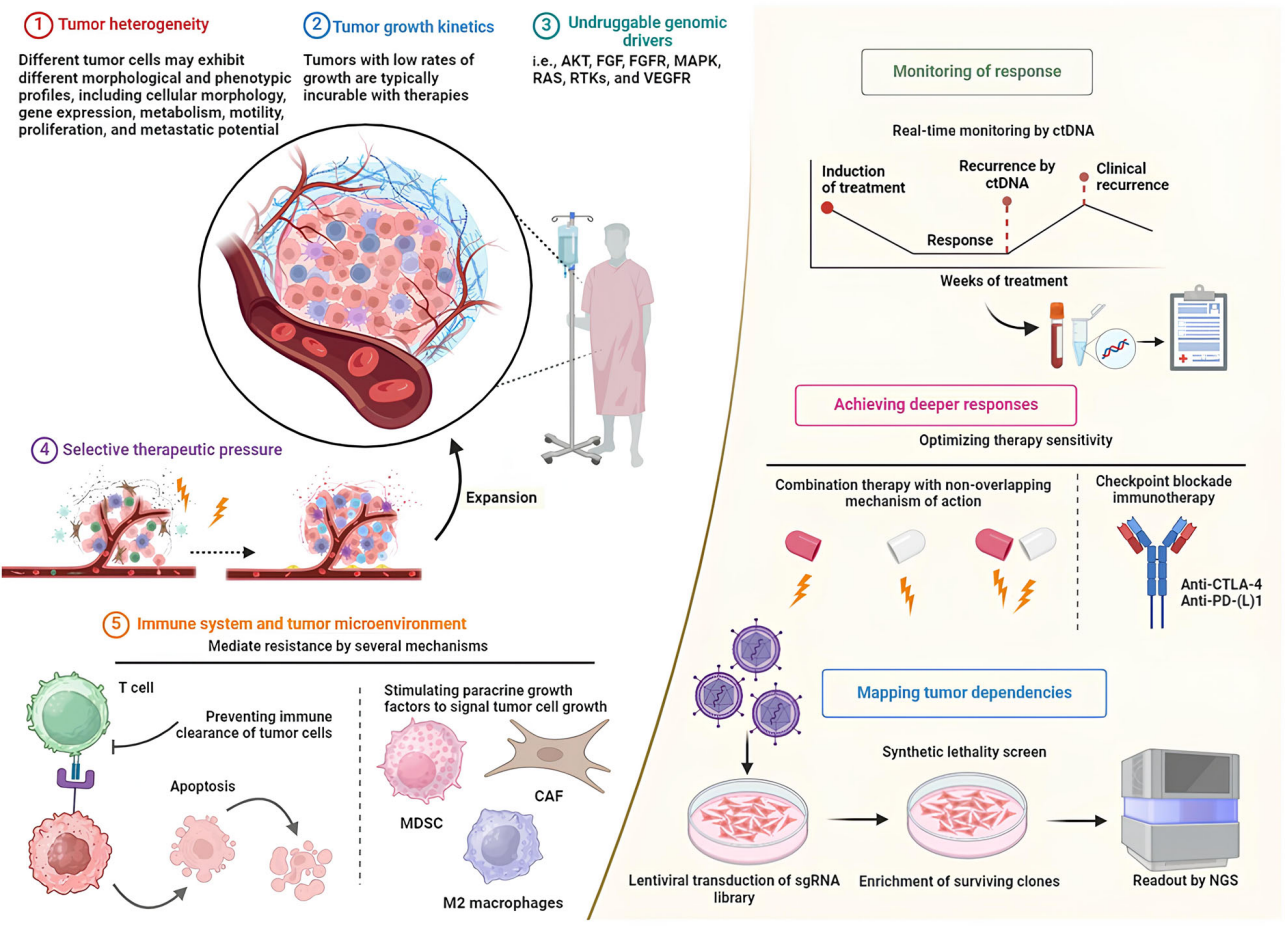
Illustration of key determinants of tumor resistance to drugs/therapy and some potential common solutions to them.

This review will delve into the various targeted drug therapies currently used in clinical practice for common intracranial tumors. It will explore the underlying molecular mechanisms that make these therapies effective, discuss the current challenges and controversies surrounding their use, and consider future directions for research and development in this field. By providing a comprehensive overview of the state of targeted drug therapy for intracranial tumors, this article aims to inform ongoing efforts to refine these treatments and to offer new perspectives on the management of these complex and life-threatening conditions (Fig. 2).

## Targeted therapy for glioma

Gliomas represent the most prevalent form of primary intracranial malignancies [2] and constitute the largest category of tumors managed within the field of neurosurgery. Currently, surgical resection remains the cornerstone of glioma treatment, with the extent of tumor removal being closely linked to patient prognosis [3]. However, due to the infiltrative nature of gliomas, distinguishing tumor margins from normal brain tissue is challenging, making it difficult to achieve complete resection in a clinical setting [4]. For glioblastoma, the most aggressive subtype of glioma, the established treatment protocol since 2005 includes surgical resection followed by fractionated radiotherapy in combination with the alkylating agent temozolomide [5]. Despite adherence to this regimen, the median overall survival for patients with glioblastoma remains disappointingly low, at approximately 15 months. This stark reality underscores the urgent need for more effective treatment strategies for this devastating malignancy. With the rapid advancements in molecular biology and genomics, the genetic mutations that drive the development and progression of gliomas are becoming increasingly understood. As a result, new targeted therapies, designed to specifically address these molecular alterations, are gaining traction and offer the potential to be more effective treatment options for glioma patients. These emerging therapies represent a promising shift towards more personalized and precise interventions in the fight against this challenging form of cancer.

### RTKs pathway inhibitors

High-grade gliomas (HGGs) encompass a group of aggressive brain tumors that include glioblastoma (GBM) as the most prevalent and severe subtype. While both HGGs and GBM share certain characteristics, such as rapid proliferation and poor prognosis, they exhibit distinct molecular profiles and clinical trial outcomes that necessitate clear differentiation in discussion and citations a grade IV glioma by the World Health Organization (WHO), is known for its resistance to conventional therapies and short median survival rate of approximately 15 months, even with a standard treatment regimen [2, 3].

**Fig. 3:**
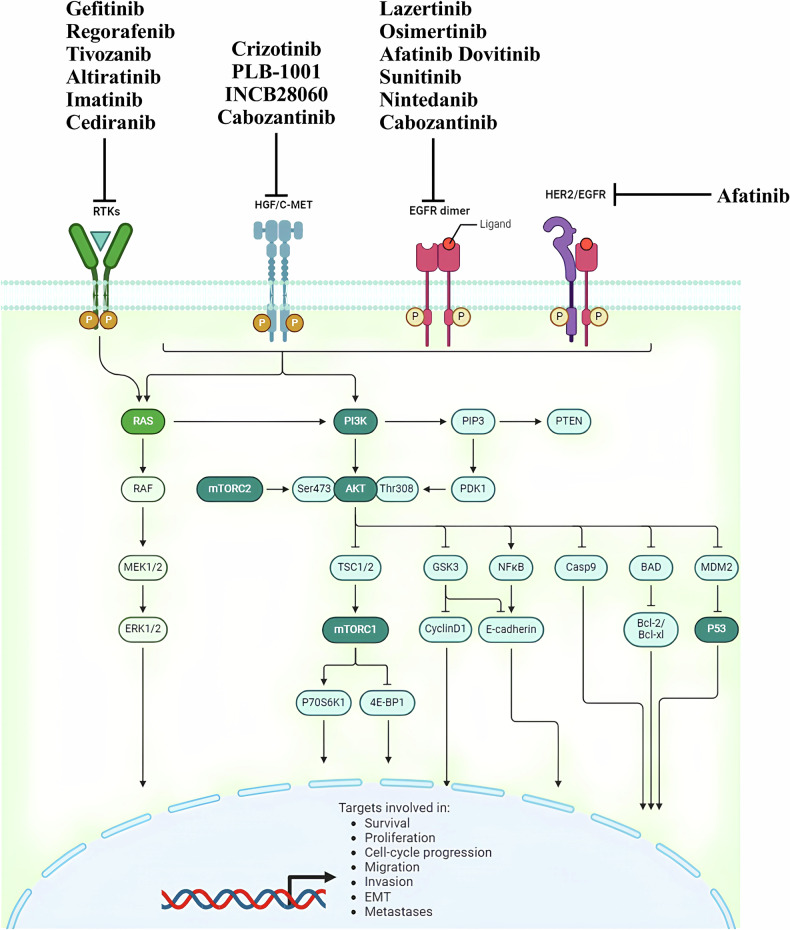
Therapies in pre-clinical and clinical phases to treat glioma with RTKs and EGFR inhibitors.

All HGGs exhibit genetic alterations in key signaling pathways, including receptor tyrosine kinases (RTKs), phosphatidylinositol-3-kinases (PI3K), and rat sarcoma (RAS) pathways [6]. RTKs function both as enzymes and receptors, encompassing platelet-derived growth factor receptor (PDGFR), epidermal growth factor receptor (EGFR), vascular endothelial growth factor receptor (VEGFR), fibroblast growth factor receptor (FGFR), among others. The overactivation of oncogenic RTKs can be driven by various mechanisms, such as ligand-independent receptor oligomerization due to gene amplification and RTK overexpression, as well as constitutive activation and ligand overexpression resulting from receptor mutations. These dysregulated RTKs play a critical role in driving oncogenic processes, including unchecked cell proliferation, abnormal survival, and the maintenance of tumor cell stemness, all of which are closely linked to tumor aggressiveness and spread [7]. In adult HGGs, the EGFR gene is the most frequently amplified, with approximately one-third of glioblastomas exhibiting EGFR gene rearrangements [8]. While EGFR inhibitors have shown efficacy in treating certain cancers, such as EGFR-mutated non-small cell lung cancer (NSCLC) [9], their effectiveness in HGGs has been disappointing. Numerous clinical trials have demonstrated that various EGFR inhibitors, including erlotinib, gefitinib, and lapatinib, whether used alone or in combination, are largely ineffective in treating HGGs [10–12]. This lack of efficacy may be attributed to several factors, such as the absence of necessary kinase domain mutations for a sustained therapeutic response, poor central nervous system drug penetration, or toxicity issues. VEGFR, expressed by vascular endothelial cells in gliomas, plays a crucial role in promoting tumor-associated angiogenesis. Bevacizumab, the most extensively studied VEGFR pathway inhibitor, received approval from the United States Food and Drug Administration (FDA) for the treatment of recurrent glioblastoma based on promising objective imaging data. However, subsequent large phase III clinical trials failed to demonstrate any significant survival benefit for patients with newly diagnosed glioblastoma [13, 14]. In contrast to the successes of RTK inhibitors in the targeted treatment of other malignancies, the outcomes of these pathway inhibitors in HGGs have been largely disappointing (Fig. 3). This highlights the complexity of targeting these pathways in gliomas and underscores the need for continued research to develop more effective therapeutic strategies.

### PI3K/AKT and mTOR pathway inhibitors

Approximately 90% of patients with glioblastoma exhibit at least one alteration in the PI3K signaling pathway. These alterations may arise from activating mutations in PI3K itself, loss of the tumor suppressor gene phosphatase and tensin homolog (PTEN), or activation downstream of RTKs [15]. The PI3K pathway’s downstream effectors include AKT and mammalian target of rapamycin (mTOR), with the mTOR complex being composed of mTORC1 and mTORC2, both of which play crucial roles in cellular metabolism, survival, and protein synthesis. Given these roles, it is theoretically plausible that targeting this pathway with specific inhibitors could be an effective strategy for treating tumors. However, clinical trials have failed to meet these expectations. First-generation mTOR inhibitors, such as rapamycin, tamsirolimus, and everolimus, have demonstrated antitumor activity as monotherapies in both in vitro and in vivo models. These inhibitors have been evaluated in multiple Phase I/II clinical trials for the treatment of newly diagnosed and recurrent HGGs. While some radiographic responses were observed in subgroups of patients with HGGs treated with everolimus or tamsirolimus, there was no significant impact on progression-free survival or overall survival when these inhibitors were used either alone or in combination with bevacizumab for recurrent glioblastoma [16–18].

**Fig. 4:**
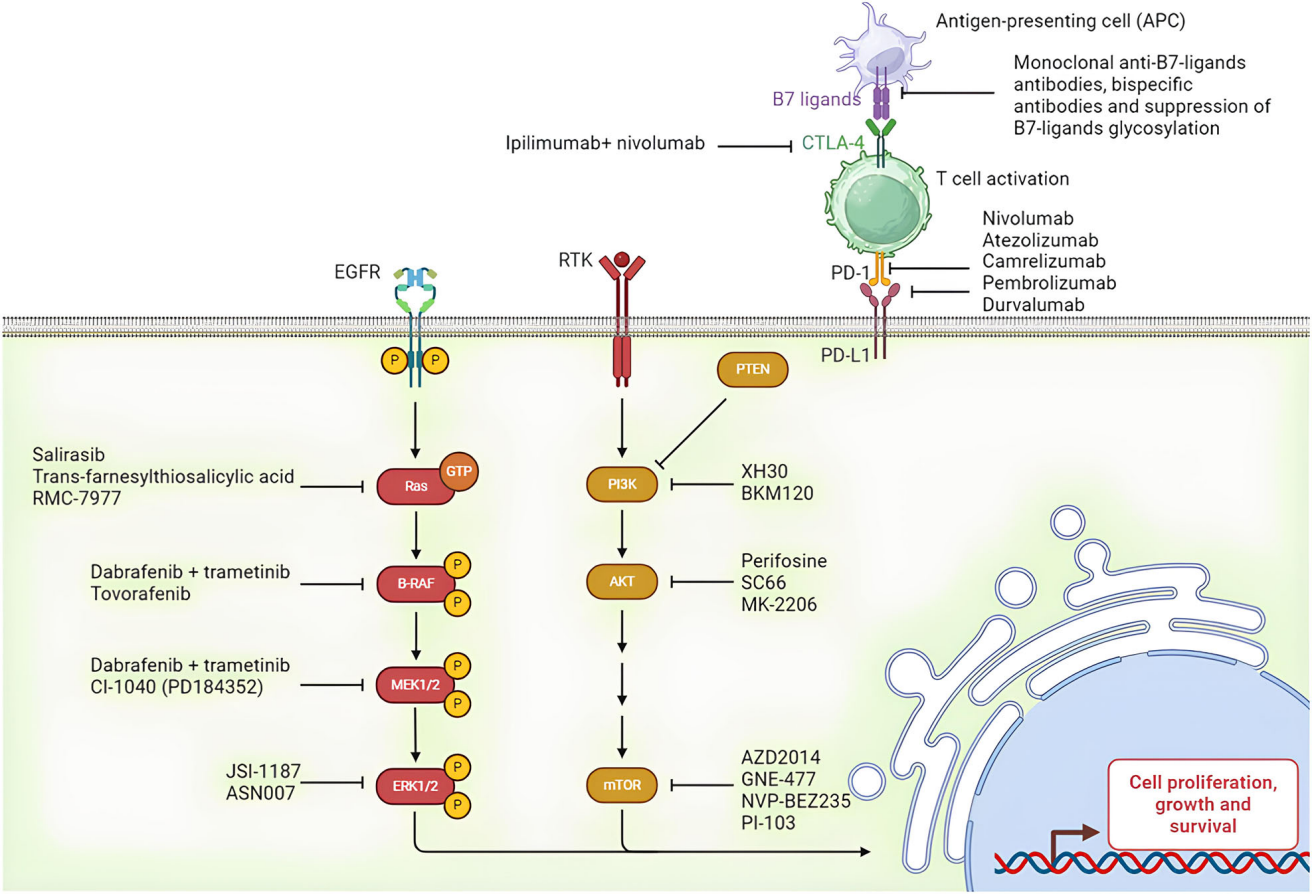
Therapies in pre-clinical and clinical phases to treat glioma with PI3K/AKT/mTOR and RAF/MEK/ERK pathway inhibitors, and immune therapy. T-cell deactivation, or tolerance, is regulated by molecules like PD-1/PD-L1 and CTLA-4 to prevent excessive immune reactions in glioma.

In discussing targeted therapies for gliomas, it is important to clarify the context in which low-grade gliomas (LGG) are mentioned to prevent confusion. While the primary focus of this review is HGGs and GBM, some ongoing clinical trials and emerging treatment strategies encompass a broader range of gliomas, including LGGs. This is because certain molecular pathways targeted in HGGs are also relevant for LGGs, thereby providing a comprehensive understanding of how these therapies can be tailored for different grades of glioma malignancy. Although the PI3K/AKT/mTOR pathway and its inhibitors are often discussed in the context of aggressive tumors such as GBMs, research may also play a significant role in the development and progression of LGGs. Ongoing trials investigating therapies like everolimus (NCT02023905) or dual mTORC1/2 inhibitors such as sapanisertib (NCT02133183) include subgroups of patients with progressive supratentorial LGGs, aiming to explore the therapeutic potential across glioma types (Fig. 4). Despite the challenges faced in earlier trials, ongoing research seeks to refine and optimize these therapeutic strategies, with the hope of eventually improving outcomes for patients with gliomas.

### RAF/MEK/ERK pathway inhibitors

Activating mutations in the mitogen-activated protein kinase (MAPK) pathway may contribute to the inhibition of HGGs, particularly in patients who harbor a point mutation or the KIAA1549 fusion, which activates the V600E mutation in the B-Raf proto-oncogene (BRAF) [19]. In a clinical trial involving gliomas with the BRAF-V600E mutation, the BRAF inhibitor dabrafenib (NCT01677741) demonstrated significant clinical activity and was well tolerated in patients with this mutation. Ongoing studies are investigating the use of the mitogen-activated protein kinase (MEK) inhibitor trametinib (NCT03919071) in combination with dabrafenib to assess the efficacy and safety of this treatment in HGGs following initial radiation therapy. One critical factor to consider is the penetration of these drugs through the blood-brain barrier, which has not been thoroughly studied for most, except dabrafenib [20]. Encouragingly, there have been individual case reports where dabrafenib has shown a favorable clinical response in pediatric patients with BRAF-V600E mutant glioblastoma (epithelioid glioblastoma) [21]. Epithelioid glioblastoma, while sharing the name and certain characteristics with conventional GBM, is a distinct subtype with unique molecular and histological features. Unlike classic GBM, E-GBM exhibits a different genetic profile, including BRAF V600E mutations, which can influence both its behavior and response to targeted therapies and present the differences between E-GBM and traditional GBM. The inclusion of E-GBM here is intended to highlight the unique treatment pathways available, such as targeted BRAF and MEK inhibition, which have shown promise in treating tumors with specific mutations.

However, a significant challenge remains: the reactivation of MAPK pathways, which limits the sustained clinical efficacy of dabrafenib and can lead to drug-related side effects [22, 23]. To address these issues, researchers have proposed combining dabrafenib with other MAPK pathway inhibitors, such as trametinib, to delay the development of resistance and reduce the adverse effects associated with BRAF inhibitors, particularly skin toxicity [24]. This combinatorial approach may enhance the therapeutic benefits and improve the overall management of HGGs with BRAF mutations (Fig. 4).

### IDH gene mutation inhibitors

**Fig. 5:**
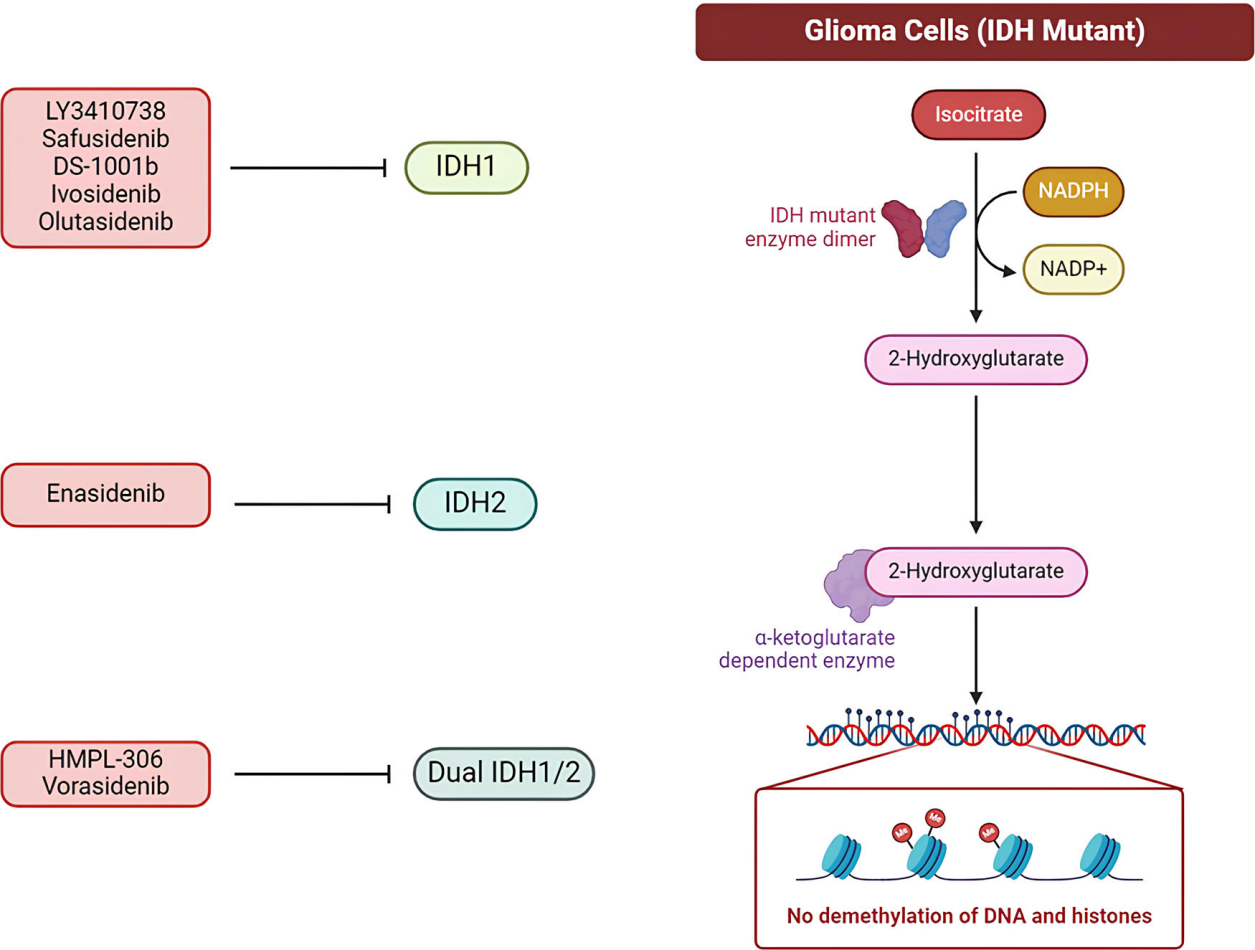
This schematic illustrates the role of IDH enzymes in glioma condition and IDH inhibitors. In glioma cells, IDH enzymes are mutated, leading to 2-hydroxyglutarate (2-HG) accumulation in the cell. 2-HG inhibits the action of alpha-ketoglutarate, leading to histone hyper-methylation and dysregulated genes.

The activation of oncogenes and the loss of tumor suppressors lead to the reprogramming of cellular metabolism, enhancing nutrient uptake and improving the energy supply within cells, thereby supporting tumor growth and survival. In malignant gliomas, the primary oncogenic mutations responsible for this metabolic reprogramming are found in the isocitrate dehydrogenase 1/2 (IDH1/2) genes, which encode IDH. Currently, there is no established treatment specifically targeting the metabolic pathway alterations caused by IDH1/2 mutations. However, several research protocols are in clinical trials and preclinical development stages. Selective inhibitors targeting mutant forms of IDH1 (AG-120), IDH2 (AG-221), or both IDH1/2 (AG-881) have progressed to clinical trials. Ivosidenib (AG-120), for example, has shown promising clinical antitumor activity in glioma patients with IDH1 mutations, and it has been well tolerated by patients, though the optimal dosing for glioma treatment is still under investigation (NCT02073994) [25]. The clinical trial of the oral IDH2 inhibitor enasidenib (AG-221) in adults has been completed, and results are awaited (NCT02273739). Additionally, Vorasidenib (AG-881) has demonstrated significant tumor shrinkage in many patients with LGGs harboring IDH mutations, indicating a strong therapeutic effect in this population [26]. However, the same drug has shown limited effectiveness in treating HGGs with IDH mutations (NCT02481154) (Fig. 5). These findings highlight the potential of IDH inhibitors in treating gliomas with specific genetic alterations, although further research is needed to optimize dosing and expand the therapeutic benefits, particularly for HGGs.

### Immune checkpoint inhibitors

Chimeric Antigen Receptor T cells (CAR T cells) represent a form of immunotherapy using genetically modified T lymphocytes, which have shown highly promising results in treating acute lymphoblastic leukemia (ALL) [27, 28]. CAR T-cells are patient-derived T cells that are transfected in vitro with a lentiviral vector to express a chimeric receptor designed to recognize tumor cells. This chimeric receptor combines the variable regions of an immunoglobulin specific to a tumor epitope with the transmembrane and cytoplasmic regions of proteins involved in T cell activation. Several CAR T-cells are currently being developed for glioblastomas, specifically targeting neo-antigens such as IL-13R-α2, EGFRvIII, Cytomegalovirus (CMV) antigens, and HER2 (Table 1).

**Table 1:** Current CAR T-cell Trials in Glioblastomas.

Target	Phase	Patient count	Sponsor	Trial Number
CMV	I	18	Medical College of Texas	NCT01123458
EGFRvIII	I	24	Beijing Brain Institute	NCT02856743
EGFRvIII	I	45	Duke University	NCT02686765
EGFRvIII	I/II	22	National Cancer Institute	NCT03212354
Her-2	I	12	University of Texas	NCT02498745
IL-13Rα2	I	95	Hope Research Center	NCT02278678
PD-L1	I	19	Beijing Brain Hospital	NCT02945667

O’Rourke et al. reported a study involving 10 patients treated with CAR T-cells directed against EGFRvIII [29]. Although the efficacy was limited, post-injection biopsies demonstrated the presence of CAR T-cells within glioblastomas, showing that CAR T-cells can cross the blood-brain barrier, making them a promising therapeutic approach for these patients. Brown et al. also highlighted the potential of CAR T-cells in a case involving a heavily pretreated 50-year-old patient. Six weekly injections were administered into the surgical cavity via a catheter, resulting in a complete response according to RANO (Response Assessment in Neuro-Oncology) criteria, cessation of corticosteroids, and a response duration of 7.5 months [30].

The main immune checkpoint inhibitors currently in clinical use are antibodies that target the Programmed Death 1 (PD-1) receptor and its ligand, Programmed Death Ligand 1 (PD-L1), thereby lifting the inhibition imposed on T lymphocytes by tumor cells. Anti-PD-1 and anti-PD-L1 agents have demonstrated efficacy across various tumor types, including melanoma, lung cancer, and kidney cancer [31–33]. In certain tumor types, PD-L1 expression is considered a predictive factor for response to anti-PD-1 or anti-PD-L1 agents. In glioblastomas, PD-L1 is expressed in 88% of newly diagnosed cases and 72% of recurrent cases [34]. High PD-L1 expression is associated with a poorer prognosis [35]. In other cancers, a high tumor mutational burden is also linked to a better response to immune checkpoint inhibitors [36]. Glioblastomas, however, have a relatively low mutational burden compared to other tumor types [37]. Nevertheless, recent studies have found that some recurrent glioblastomas acquire deficiencies in the Mismatch Repair (MMR) system, with 26% of recurrent tumors showing MSH6 mutations after treatment with temozolomide and radiotherapy [38–40]. This mutation acquisition after initial treatment could sensitize these tumors to immune checkpoint inhibitors.

Numerous studies have evaluated anti-PD-1 agents in recurrent glioblastoma patients (NCT02017717, NCT02336165, NCT02337491, NCT02054806) (Table 2). Response rates in this setting range from 2.5% to 13.3% for anti-PD-1 or anti-PD-L1 monotherapy. These studies reported six-month progression-free survival (PFS) rates between 16% and 44%, with encouraging overall survival (OS) medians between 7 and 14 months, and in some cases, median survival was not reached. One of the initial studies was the phase I CheckMate-143 study (NCT02017717), which investigated the efficacy of an anti-PD-1 agent, nivolumab, with or without the anti-CTLA4 agent ipilimumab, in patients with recurrent glioblastoma. Patients were randomized (1:1) to receive either nivolumab 3 mg/kg every 2 weeks (Q2W; NIVO3; *n* = 10) or nivolumab 1 mg/kg plus ipilimumab 3 mg/kg every three weeks for four doses, followed by nivolumab 3 mg/kg Q2W (NIVO1 + IPI3; *n* = 10). A third cohort was added, in which patients received nivolumab 3 mg/kg plus ipilimumab 1 mg/kg Q3W for four doses, followed by nivolumab 3 mg/kg Q2W (NIVO3 + IPI1; *n* = 20), based on melanoma studies suggesting that NIVO3 + IPI1 is better tolerated than NIVO1 + IPI3. In total, eight patients (20%) experienced a stable disease for 12 weeks or longer (NIVO3, *n* = 2; NIVO1 + IPI3, *n* = 2; NIVO3 + IPI1, *n* = 4), and three patients (7.5%) had a partial response (NIVO3, *n* = 1; NIVO3 + IPI1, *n* = 2). The median PFS was 1.9 months with NIVO3, 1.5 months with NIVO1 + IPI3, and 2.1 months with NIVO3 + IPI1. Median OS was 10.4 months in the NIVO3 group, 9.2 months in the NIVO1 + IPI3 group, and 7.3 months in the NIVO3 + IPI1 group. The combination of NIVO1 + IPI3 was more toxic than NIVO3 + IPI1, with 90% grade 3-4 toxicity compared to 30% [41].

**Table 2:** Open immunotherapy trials in glioblastomas.

Trial name	Target	Phase	Indication	Drug	Trial number
Anti-PD-1/PD-L1					
Neo-adjuvant Nivolumab in Glioblastoma (Neo-nivo)	Anti-PD-1	II	Primary/Recurrent GBM	Nivolumab	NCT02567843
Pharmacodynamics of Pembrolizumab in Recurrent Glioblastoma	Anti-PD-1	II	Recurrent GBM	Pembrolizumab	NCT02387645
Anti-CTLA4					
Tremelimumab + Durvalumab in Malignant Glioma	Anti-CTLA4 and Anti-PD-1	II	Recurrent GBM	Tremelimumab, Durvalumab	NCT02798743
Tumor-Focused Therapies					
Pembrolizumab + Laser Ablation in Malignant Gliomas	Anti-PD-1	I/II	Recurrent GBM	Pembrolizumab	NCT02367890
Immune Therapy Combinations					
Anti-LAG-3 with Urelumab and Nivolumab	Anti-LAG3 and CD137 Agonist	I	Recurrent GBM	Nivolumab, Urelumab	NCT02643210
Targeted Therapy Combinations					
Nivolumab + Bevacizumab	Anti-PD-1 and Anti-VEGF	II	Recurrent GBM	Nivolumab, Bevacizumab	NCT03456590
Combination of Adenovirus + Pembrolizumab	Anti-PD-1 and Oncolytic Virus	II	Recurrent GBM	Pembrolizumab	NCT02799788

**Fig. 6:**
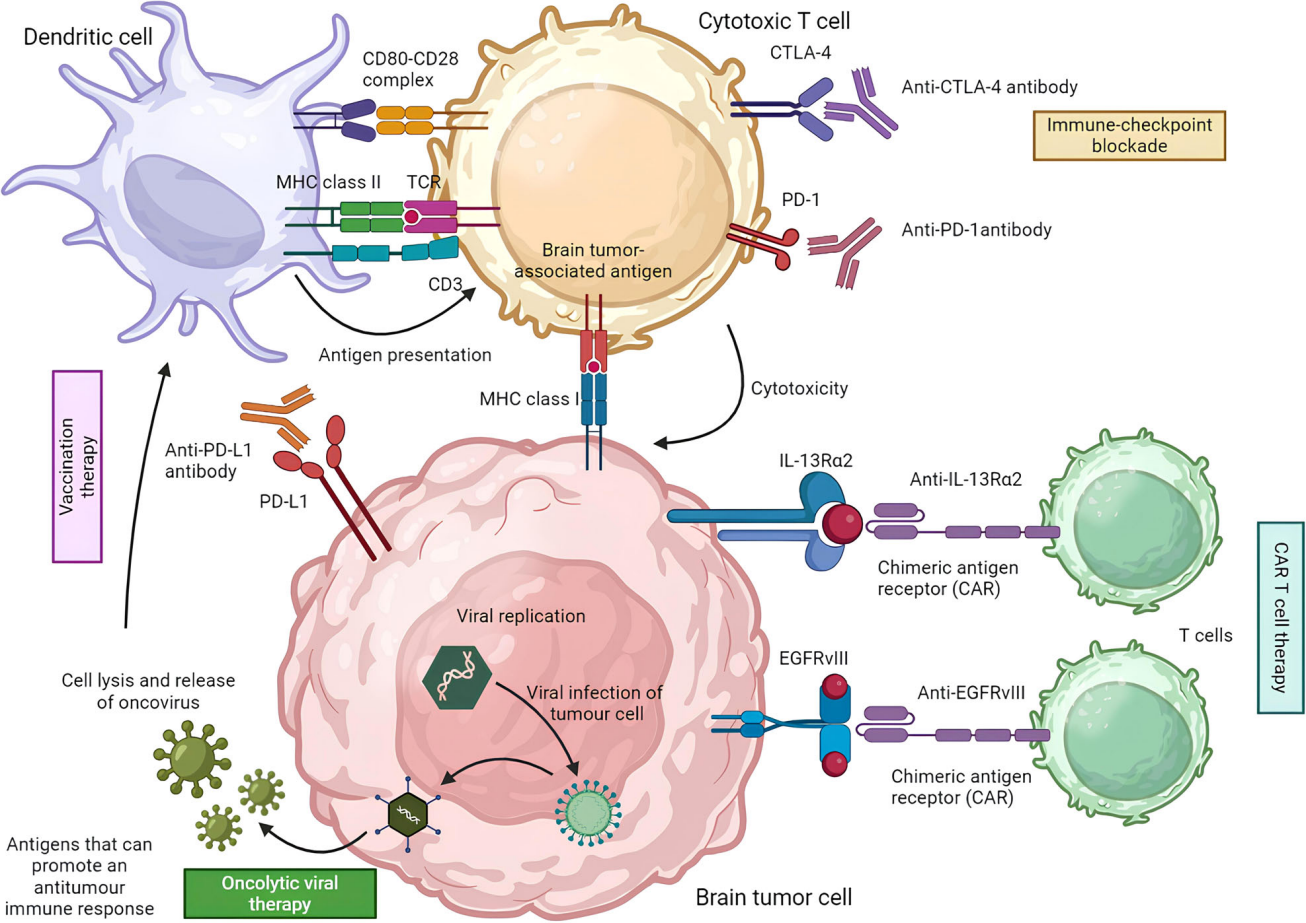
Basic principles of immunotherapy of brain tumors.

**Fig. 7:**
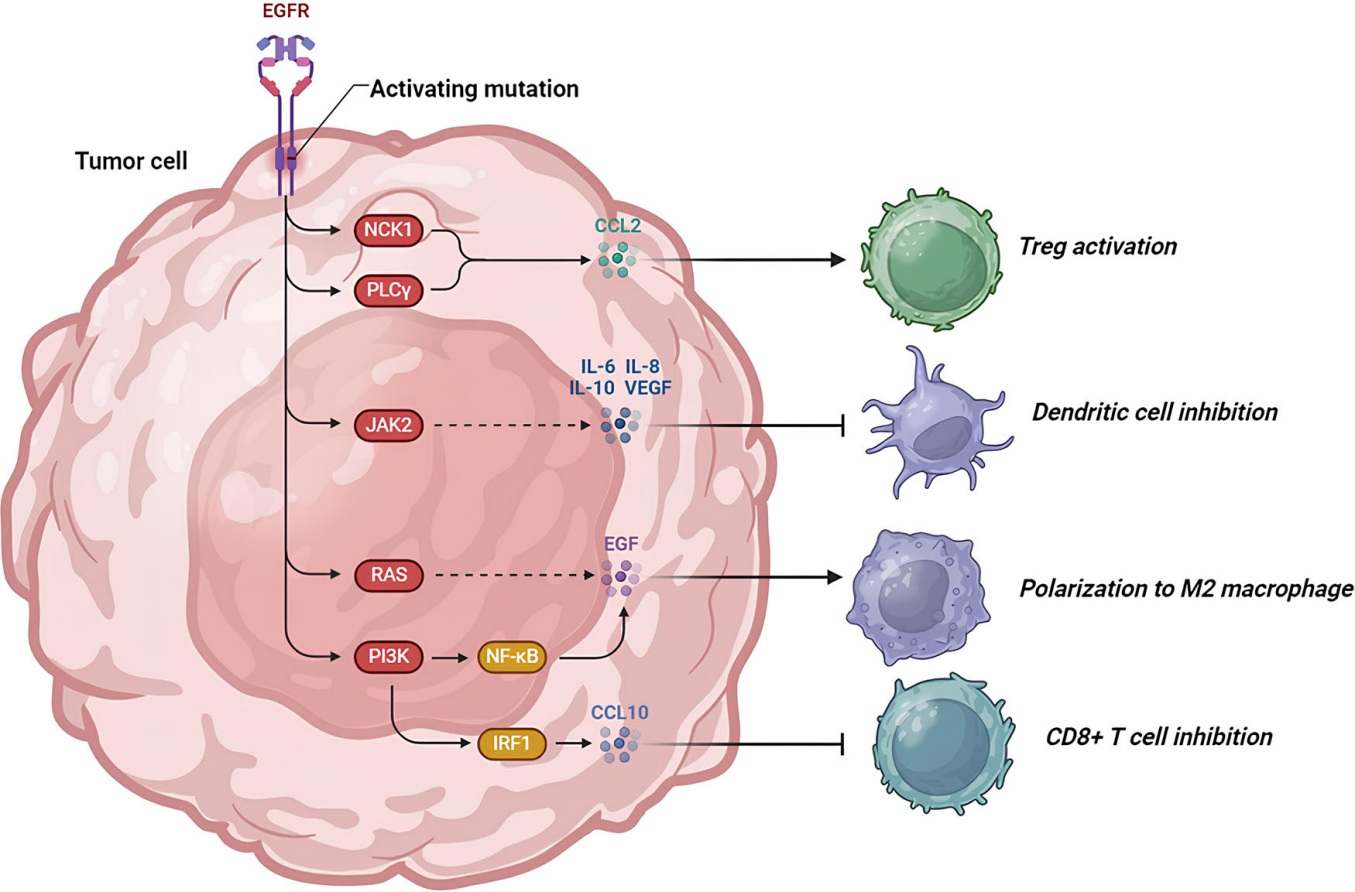
EGFR Signaling and immune stimulations in brain tumors. This illustration demonstrates how EGFR signaling in tumor cells can influence immune cells in the tumor microenvironment.

The phase III CheckMate-143 trial compared nivolumab 3 mg/kg (*n* = 184) with bevacizumab 10 mg/kg (*n* = 185). Twelve-month OS rates were comparable at 42%, with median OS of 9.8 months for nivolumab and 10 months for bevacizumab. Grade 3-4 toxicity rates were similar (18% for nivolumab vs. 15% for bevacizumab) [42]. Among patients treated with nivolumab, 8% showed sustained responses over time, with a median radiological response duration of 11 months in the nivolumab group compared to 5.3 months in the bevacizumab group among responders. The Keynote-028 study (NCT02054806) explored the efficacy of the anti-PD-1 agent pembrolizumab across multiple advanced solid tumor types, including a glioblastoma cohort (*n* = 26). In this study, one patient achieved a partial response (4%), and 12 patients (48%) had a stable disease, with a median PFS of 2.8 months and a median OS of 14.4 months [43]. Durvalumab, an anti-PD-L1 agent, has also been evaluated in patients with recurrent glioblastomas in a phase II study, both as monotherapy and in combination with bevacizumab or radiotherapy. In total, 4 patients (13.3%) had a partial response, and 14 patients (46.7%) had stable disease, with a six-month PFS rate of 20% (Figs. 4, 6 and 7) [44].

These studies highlight the need to identify predictive factors for response, particularly since responders exhibit prolonged responses. Additionally, other therapeutic approaches are under investigation, notably combination therapies (Table 3) [45].

**Table 3:** Various targeted therapies for gliomas, summarizing their mechanisms, clinical findings, limitations, emerging strategies, and future directions.

Therapy/Target	Mechanism of action	Clinical findings	Limitations/challenges	Emerging strategies	Future directions	Ref.
RTKs pathway inhibitors	Inhibit receptor tyrosine kinases (RTKs) like EGFR, VEGFR, which are involved in cell proliferation and angiogenesis.	EGFR inhibitors (e.g., erlotinib, gefitinib) largely ineffective in HGGs. Bevacizumab approved for recurrent glioblastoma due to imaging improvements but no survival benefit in Phase III trials.	Poor CNS drug penetration, absence of necessary mutations for therapeutic response, toxicity issues.	Development of next-generation inhibitors with better CNS penetration and specificity for mutated RTKs.	Continued research to develop inhibitors that can cross the blood-brain barrier effectively and target specific mutations. Potential for combinational therapies to overcome resistance mechanisms.	[6–14]
PI3K/AKT/mTOR pathway inhibitors	Target PI3K pathway alterations affecting downstream effectors like AKT and mTOR, critical for cell survival and metabolism.	First-gen mTOR inhibitors (e.g., everolimus) demonstrated antitumor activity in vitro/in vivo but no significant impact on survival in clinical trials. Dual inhibitors (e.g., sapanisertib) currently under investigation.	Limited clinical efficacy, need for improved drug formulations for better CNS penetration, and optimized combinations for enhanced therapeutic effect.	Exploring combination therapies involving PI3K/AKT/mTOR inhibitors with other targeted agents or immunotherapies to enhance efficacy.	Need to understand better the molecular determinants of response to guide personalized treatment approaches. Trials exploring dual inhibition (e.g., sapanisertib) and combinations with other modalities.	[15–18]
RAF/MEK/ERK pathway inhibitors	Target MAPK pathway mutations, particularly BRAF-V600E, involved in tumor growth.	BRAF inhibitor dabrafenib effective in gliomas with BRAF-V600E mutation; combination with MEK inhibitor trametinib being tested to enhance efficacy and reduce resistance.	Challenges with blood-brain barrier penetration, potential for drug resistance, and side effects such as skin toxicity.	Investigating drug combinations to delay resistance development, particularly using MEK inhibitors alongside BRAF inhibitors.	Research into overcoming blood-brain barrier challenges and minimizing side effects while maintaining efficacy.	[19–24]
IDH Gene mutation inhibitors	Inhibit mutant IDH1/2 enzymes, which reprogram tumor metabolism and contribute to glioma growth.	IDH inhibitors (e.g., ivosidenib, vorasidenib) showing promise in low-grade gliomas (LGGs) with IDH mutations; ongoing trials to assess dosing and effectiveness in high-grade gliomas (HGGs).	Limited effectiveness in HGGs, need for further research on optimal dosing and combination therapies to improve outcomes.	Ongoing trials to refine dosing and evaluate the combination of IDH inhibitors with other targeted therapies or standard treatments like temozolomide.	Continued exploration of combination strategies and biomarker development to identify patients most likely to benefit. Focus on expanding efficacy beyond low-grade gliomas to more aggressive types.	[19, 24]
Immune checkpoint inhibitors	Block immune evasion mechanisms, such as PD-1/PD-L1 pathways, to enhance T-cell-mediated tumor destruction.	CAR-T cell therapies have shown potential in glioblastoma, extending survival in some patients; however, no phase III results yet.	High recurrence rates, incomplete understanding of optimal targets and delivery mechanisms, and limited penetration into tumor sites.	Optimizing CAR-T cell design and identifying novel immune checkpoint targets to improve efficacy and safety.	Further clinical trials to establish the safety and efficacy of CAR-T cells in larger, more diverse patient populations. Research into enhancing CAR-T cell penetration and retention in brain tumors.	[25–27]
TGF-β receptor inhibitors	Inhibit TGF-β, a key regulator that suppresses T-cell function within the tumor microenvironment and contributes to therapeutic resistance.	Galunisertib, a TGF-β receptor kinase inhibitor, was not effective in glioblastoma in trials; potential in combination with temozolomide to overcome resistance and improve outcomes.	Limited efficacy as monotherapy, need for combination approaches to enhance therapeutic response and overcome drug resistance.	Investigating combination therapies with TGF-β inhibitors and other standard treatments like temozolomide to address resistance mechanisms.	Future studies to explore biomarkers for TGF-β inhibitor response and to identify patient subsets that may benefit most. Trials combining TGF-β inhibitors with immune modulators or chemotherapy agents.	[28–30]
Cytokine therapy	Utilize cytokines like ILs and IFNs to boost immune responses or alter tumor microenvironment to suppress tumor growth.	IL-2, IL-4 gene vaccine, and IFN-α demonstrated positive responses in early trials; however, IFN-γ did not show significant benefits in combination with standard therapy for glioblastoma.	Variable efficacy depending on cytokine type, potential for significant side effects, and need for further trials to determine optimal combinations and dosing.	Developing cytokine-based combination therapies to synergize with existing standard of care or novel targeted therapies.	Continued investigation into the specific cytokine pathways involved in glioma immune modulation. Exploration of gene editing technologies to enhance cytokine-based therapies.	[31–36]

### Transforming growth factor (TGF) receptor inhibitors

Transforming Growth Factor Beta (TGF-β) is a multifunctional family of proteins that regulate cell proliferation, differentiation, and immune responses. TGF-β has three isoforms: TGF-β1, TGF-β2, and TGF-β3, each playing distinct roles in cellular processes. In the context of glioblastoma, TGF-β2 is particularly significant due to its high expression levels in approximately 90% of glioblastoma cellss known for promoting immune suppression within the tumor microenvironment, which can contribute to tumor growth and resistance to treatments [46–48].

TGF-β protein family plays a crucial role in various regulatory pathways and acts as a significant T cell inhibitor within the glioblastoma tumor microenvironment. Notably, TGF-β2 is expressed in approximately 90% of glioblastoma cells [46]. While TGF-β2 inhibitors have been utilized in the treatment of other types of cancer, their application as therapeutic targets in glioblastoma remains challenging. A clinical study comparing the efficacy of the TGF-β receptor kinase inhibitor galunisertib with Lumostine (NCT01582269) in treating glioblastoma revealed that this treatment approach was not effective for this cancer [47]. Recent research has identified a connection between TGF-β and resistance to temozolomide, as well as the expression of methylguanine methyltransferase (MGMT) [48]. These findings suggest that combining TGF-β inhibitors with temozolomide could represent a novel therapeutic strategy for inhibiting glioblastoma progression. Further investigation into this combination therapy may offer new insights into overcoming the challenges associated with treating this aggressive form of brain cancer.

### Cytokine therapy

Cytokines produced within the immune microenvironment of tumor cells can be exploited by tumors to either suppress or induce immune responses [49]. Among these cytokines, interleukins (ILs) and interferons (IFNs) are the most widely studied and utilized in cancer treatment. Research into the use of IL-2 in glioma patients began as early as 1986, and a phase I trial investigating a glioma cell vaccine transfected with a gene encoding IL-4 showed promising clinical responses in patients with HGGs [50]. Additionally, two phase II clinical trials combining temozolomide with IFN-α demonstrated that this combination was effective in treating glioblastoma, with patients responding well to the regimen [51]. Phase I and phase II clinical trials primarily aim to evaluate the safety, tolerability, and optimal dosing of treatments rather than focusing on survival outcomes. The trials involving IFN treatments for glioblastoma, such as those combining temozolomide with IFN-α and IFN-β, demonstrated that these regimens were generally safe and well-tolerated [51, 52]. Although some preliminary clinical responses were noted, these phases are not designed to definitively establish survival benefits. For instance, phase I trials often assess adverse events and determine the maximum tolerated dose, while phase II trials focus on the initial efficacy signals and further safety profiling [52, 53]. Therefore, while these studies have shown that IFN therapies are feasible and safe, more comprehensive phase III trials are needed to robustly evaluate their impact on overall survival and long-term outcomes. However, the combination of IFN-γ with standard chemoradiotherapy has not shown clinical benefits for glioblastoma patients (Table 3) [54].

## Targeted therapy for meningioma

Meningiomas originate from the arachnoid cap cells of the leptomeninges and are the second most common type of primary tumor within the central nervous system [55]. Approximately 80% to 90% of meningiomas are benign (classified as WHO grade 1) and can often be effectively managed with long-term routine follow-up, surgical resection, or radiation therapy [56]. However, atypical meningiomas (WHO grade 2) and anaplastic meningiomas (WHO grade 3, also referred to as “malignant meningiomas”) present significant treatment challenges, as they are not typically responsive to surgery, radiotherapy, or conventional chemotherapy. While drug therapy has traditionally played a limited role in the treatment of meningiomas, targeted drug therapy offers a promising non-invasive alternative for managing meningiomas that are resistant to conventional treatments. This emerging approach provides new hope for patients with these more aggressive forms of meningioma, where traditional methods have proven inadequate.

### RTKs pathway inhibitors

**Fig. 8:**
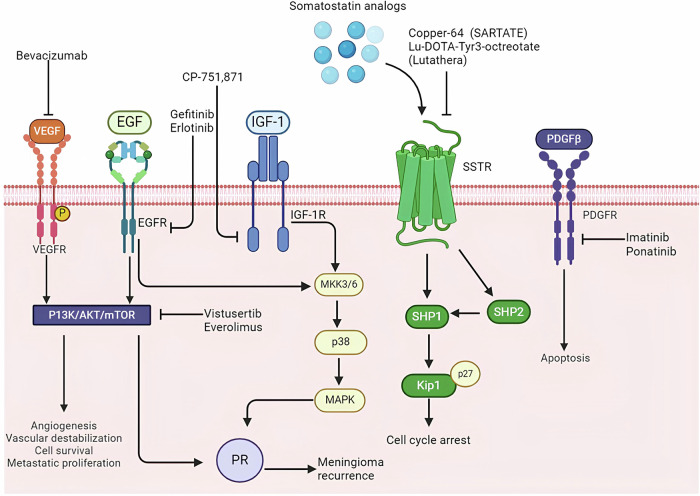
Therapies in pre-clinical and clinical phases to treat meningioma. The growth factor receptor signaling is stimulated by its ligands (e.g. EGF and IGF-1), resulting in the activation of MAPK-related pathways and the subsequent stimulation of both (1) the ligand independent receptor activation and (2) the AKT/mTOR pathway, which increases PR expression, respectively. In addition, the activation of SSTR promote cell cycle progression.

Overexpression of RTKs has been observed in malignant meningiomas, leading to a growing interest in using RTK inhibitors for targeted therapy of these tumors [57]. Among RTKs, the overexpression of PDGFR is particularly associated with the development of malignant and atypical meningiomas. In a study involving 21 patients with recurrent or invasive meningiomas treated with the PDGFR inhibitor imatinib in combination with hydroxyurea, 67% of the patients showed no tumor progression on imaging. Although this combination therapy was well tolerated, it had limited efficacy in treating WHO grade 2 or 3 meningiomas [58]. Sunitinib, a small molecule tyrosine kinase inhibitor targeting both VEGFR and PDGFR, has also been investigated for its potential in treating malignant meningiomas. A prospective, multicenter, single-arm Phase II clinical trial demonstrated that 42% of patients treated with sunitinib did not experience tumor progression within six months. Furthermore, magnetic resonance perfusion imaging indicated that sunitinib effectively reached the tumor site and exerted effects on the tumor’s vascular system. However, these findings require further clinical validation [59]. In addition to PDGFR, overexpression of EGFR has been identified in more than 60% of meningiomas [60]. Despite this, a study involving 25 patients with recurrent meningioma treated with a combination of EGFR inhibitors gefitinib and erlotinib found no significant clinical response. Although the treatment was well tolerated, the lack of efficacy suggests that EGFR alone may not be a sufficient target for meningioma therapy. This underscores the need to explore the therapeutic potential of combining multi-target inhibitors with EGFR inhibitors to achieve better outcomes [61]. Moreover, VEGF has been found to be expressed in 84% of meningiomas, with its expression levels increasing in correlation with the tumor grade [62]. The VEGF inhibitor bevacizumab has shown clinical benefits in patients with meningiomas that are refractory to both surgery and radiation therapy [63]. However, the current evidence on the survival benefits and the potential drug-related toxicities is insufficient, highlighting the need for further evaluation of bevacizumab’s efficacy in treating meningiomas. Randomized controlled trials are particularly necessary to fully understand the role of bevacizumab in the management of meningiomas [64]. These findings collectively point to the complexity of targeting specific pathways in meningioma treatment and suggest that a multi-targeted approach may be necessary to achieve more effective therapeutic outcomes (Fig. 8).

### PI3K/AKT and mTOR pathway inhibitors

The PI3K/AKT/mTOR pathway is a crucial signaling cascade involved in cellular growth, survival, and metabolism. It begins with the activation of phosphatidylinositol 3-kinase (PI3K), which, when triggered by upstream signals such as receptor tyrosine kinases (RTKs), converts PIP2 to PIP3. This conversion leads to the recruitment and activation of AKT (also known as protein kinase B). Once activated, AKT phosphorylates a range of downstream targets, including the mammalian target of rapamycin (mTOR), which exists in two complexes: mTORC1 and mTORC2. These complexes regulate key processes like protein synthesis, cellular proliferation, and survival. Alterations in the PI3K/AKT/mTOR pathway, such as mutations or loss of the tumor suppressor PTEN, are frequently observed in many cancer types, including gliomas. This pathway’s hyperactivation is associated with increased tumor growth and resistance to conventional therapies. Consequently, inhibitors targeting various components of this pathway, including PI3K inhibitors, AKT inhibitors, and mTOR inhibitors, have been explored for their therapeutic potential. However, clinical outcomes have varied, highlighting the complexity of effectively targeting this pathway without significant side effects or resistance mechanisms [65]. Recently, PIK3CA mutations have been identified in a significant number of skull base lesions [65]. Tumors located at the base of the skull are particularly challenging and risky to treat with surgery or radiation therapy, making targeted therapies that focus on the PI3K/AKT/mTOR pathway a potential new treatment option for patients with skull base meningiomas. However, recent clinical findings highlight the complexity of treating these tumors. For instance, a case report involving a patient with meningioma showed a poor response to the AKT inhibitor capivasertib, despite multiple surgical resections, radiation therapy, and other systemic treatments [66]. Within the mTOR pathway, mTORC1 can dampen RTK signaling through the PI3K and AKT pathways, thereby establishing a negative feedback loop. Inhibitors of the mTOR pathway, such as temsirolimus and everolimus, have demonstrated efficacy in inhibiting meningioma growth [67]. Furthermore, a Phase II clinical trial (NCT03071874) has shown that Vistusertib (AZD2014), a dual mTOR inhibitor, can slow the growth rate of meningiomas in patients with recurrent WHO grade 2 and 3 meningiomas. These findings suggest that targeting the PI3K/AKT/mTOR pathway could provide a promising therapeutic approach for difficult-to-treat meningiomas, particularly those located at the skull base (Fig. 8).

### Hormone receptor antagonists

The presence of the progesterone receptor (PR) is considered a favorable prognostic indicator for meningioma, as PR status is inversely associated with tumor grade, recurrence rates, and the mitotic index. PR expression has been observed in approximately 70% of meningioma patients (Fig. 8) [68]. To explore the potential of mifepristone as a treatment option for meningiomas that cannot be surgically removed, the United States Oncology Association conducted a multicenter, prospective, randomized, controlled Phase III trial. The results indicated that, while patients well tolerated long-term administration of mifepristone, it did not lead to improved clinical survival outcomes in those with unresectable meningiomas [68].

### Somatostatin receptor antagonists

Somatostatin (SST) plays a crucial role in regulating the proliferation of both normal and tumor cells. Long-acting SST analogs are recommended for the systemic treatment of recurrent meningiomas that cannot be fully resected or are resistant to radiation therapy (Fig. 8) [69]. A recent study investigated the efficacy of combining the SST receptor antagonist octreotide with everolimus in treating recurrent meningioma. The results showed promising outcomes, with survival rates of 90% at 6 months and 75% at 12 months for patients receiving the combination therapy. Notably, after 3 months of active treatment, 78% of patients experienced a significant reduction in tumor growth rate, with more than a 50% decrease in tumor volume. This clinical research demonstrated that the combination of octreotide and everolimus exhibits strong anti-meningioma activity (Table 4) [70].

**Table 4:** Various targeted therapies for meningiomas, summarizing their mechanisms, clinical findings, limitations, emerging strategies, and future directions.

Therapy/Target	Mechanism of action	Clinical findings	Limitations/challenges	Emerging strategies	Future directions	Ref.
RTKs pathway inhibitors	Inhibit overexpressed receptor tyrosine kinases (RTKs) such as PDGFR, VEGFR, and EGFR, which are involved in tumor growth and angiogenesis.	Imatinib + hydroxyurea showed limited efficacy in WHO grade 2 or 3 meningiomas. Sunitinib showed promise in inhibiting tumor progression in some patients. EGFR inhibitors (gefitinib, erlotinib) were well tolerated but ineffective. Bevacizumab showed benefits in refractory cases.	Limited efficacy as monotherapy; need for better understanding of multi-target approaches to improve outcomes. Potential toxicities and unclear survival benefits for some agents like bevacizumab.	Development of multi-targeted inhibitors combining RTK inhibition with other pathways; combining bevacizumab with other agents to assess efficacy.	Conduct randomized controlled trials to fully understand the benefits and limitations of multi-targeted approaches and refine combination therapies for better efficacy and safety.	[39–46]
PI3K/AKT/mTOR pathway inhibitors	Target the PI3K/AKT/mTOR pathway, which plays a crucial role in cell proliferation, invasion, and metastasis, especially in difficult-to-treat skull base meningiomas.	Case reports indicate poor response to AKT inhibitor capivasertib in some patients. mTOR inhibitors (temsirolimus, everolimus) showed efficacy in growth inhibition. Vistusertib demonstrated potential in slowing growth in recurrent grade 2/3 meningiomas.	Complex response patterns with some agents showing limited clinical impact; difficulty in targeting skull base meningiomas due to location and poor drug penetration.	Combination therapies targeting multiple nodes within the PI3K/AKT/mTOR pathway to enhance efficacy; exploring dual mTOR inhibitors like Vistusertib.	Further research to identify patient subgroups most likely to benefit from pathway inhibitors and refine combination therapies with other systemic treatments.	[47–49]
Hormone receptor antagonists	Antagonize hormone receptors such as the progesterone receptor (PR), which is inversely correlated with tumor aggressiveness and recurrence rates.	Mifepristone, a PR antagonist, was well tolerated in long-term use but did not improve clinical survival outcomes in a Phase III trial for unresectable meningiomas.	Lack of significant clinical benefit in survival despite good tolerance; need for better understanding of hormonal influence on meningioma progression.	Exploration of other hormone receptor targets or combination with other therapies to enhance effectiveness.	Further studies to investigate combination strategies with hormone receptor antagonists and identify other potential hormonal targets for therapeutic intervention.	[50]
Somatostatin receptor antagonists	Target somatostatin receptors (SST) to regulate tumor cell proliferation and enhance anti-tumor responses.	Combination of octreotide (SST antagonist) with everolimus showed promising results, with high survival rates and significant tumor volume reduction in recurrent meningioma patients.	Need for further validation in larger cohorts; potential for resistance development; limited understanding of long-term outcomes.	Development of long-acting SST analogs in combination with other targeted agents to enhance anti-tumor efficacy.	Conduct large-scale trials to establish the efficacy and safety of SST receptor antagonists combined with other treatments in diverse meningioma patient populations.	[51, 52]

## Targeted therapy for pituitary tumors

**Fig. 9:**
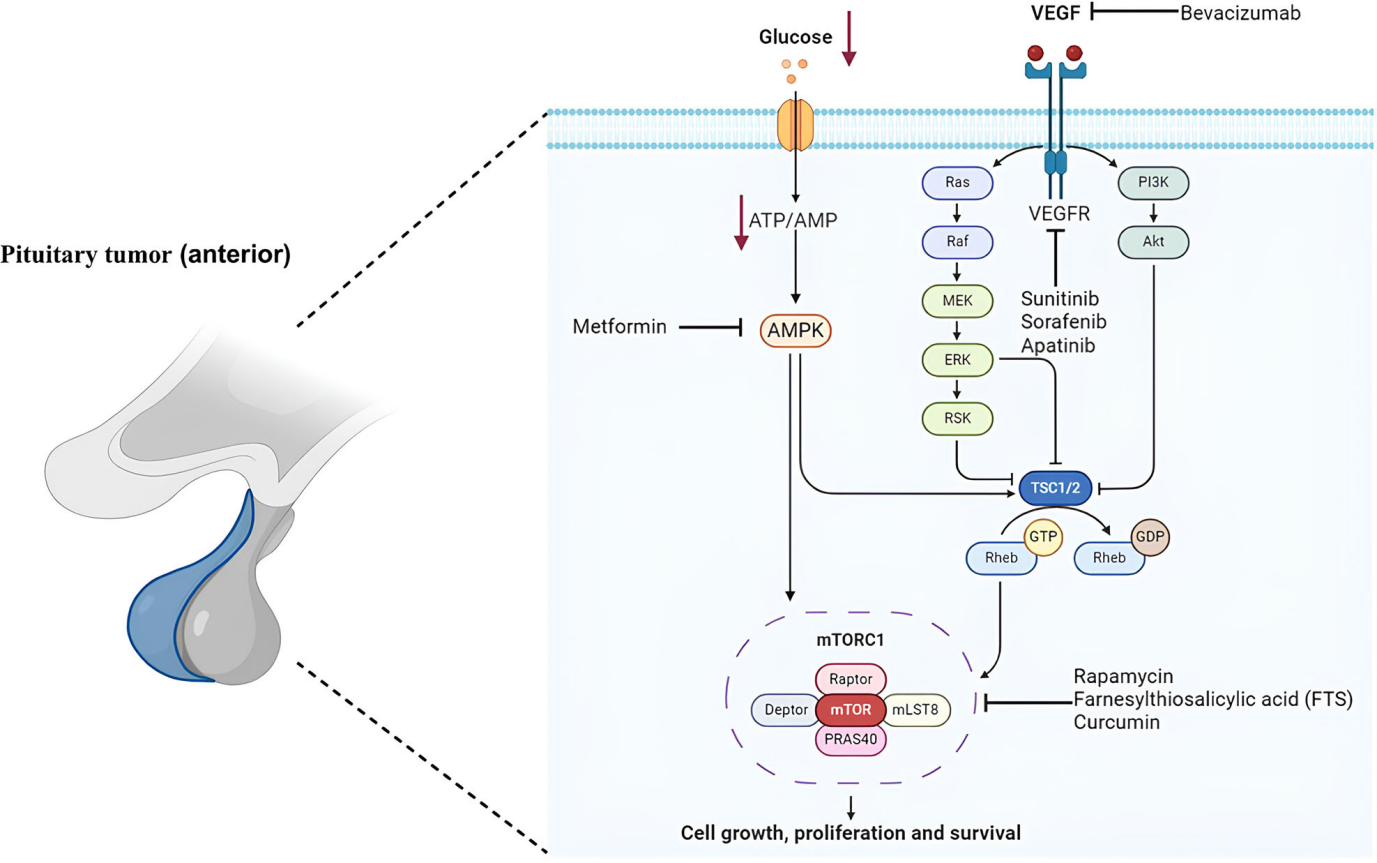
Therapies in pre-clinical and clinical phases to treat pituitary tumor (anterior). One of the major hubs of glucose metabolism is mechanistic target of rapamycin (mTOR) kinase, which forms the mTORC1 protein complex. When glucose levels are low, mTORC1 is inhibited, in turn leading to the repression of numerous anabolic processes, sparing ATP and antioxidants. The major energy sensor AMP-activated protein kinase (AMPK), as well as other independent players.

Pituitary tumors rank as the third most common intracranial tumor in adults, accounting for approximately 15% of all central nervous system tumors [71]. While most pituitary tumors are benign and can be effectively treated with surgery, a small subset exhibits aggressive behavior and may recur even after surgical resection and radiation therapy. Although temozolomide is currently the most extensively studied drug for treating aggressive pituitary adenomas and pituitary carcinomas, about 30% of patients undergoing temozolomide treatment experience disease progression. Furthermore, for those patients whose tumors initially respond to treatment, there is a tendency for the tumors to regrow once temozolomide is discontinued [72]. In addition, pituitary tumors are often accompanied by impaired glucose tolerance or diabetes mellitus, which is often an early manifestation of these tumors. Furthermore, targeting molecules in amino acid metabolic and glucose signaling pathway also has the potential to pituitary tumors. For example, targeting mTOR can control the growth of pituitary tumors (Fig. 9). Given the success of targeted therapies in treating other types of tumors, there is growing interest among medical professionals in exploring targeted drug therapies for managing aggressive pituitary tumors.

### RTKs pathway inhibitors

Aggressive pituitary tumors and pituitary cancers have been found to exhibit higher vessel density and increased VEGF expression compared to benign pituitary tumors, indicating that VEGF and angiogenesis may play a significant role in the progression of anterior pituitary tumors (Fig. 9) [73]. In a related study, seventeen patients with aggressive pituitary tumors and pituitary cancer were treated with the VEGF receptor inhibitor bevacizumab, with some also receiving a combination of bevacizumab and temozolomide. The outcomes revealed that one patient achieved complete radiographic remission, four patients showed partial remission on imaging, seven patients maintained stable disease, and three patients experienced disease progression. Additionally, two patients developed complications, specifically epistaxis and nephritis [74].

### PI3K/AKT and mTOR pathway inhibitors

The PI3K/AKT/mTOR pathway has been identified as upregulated and/or overactivated in anterior pituitary tumors, and inhibitors targeting this pathway have demonstrated antitumor effects both in vitro and in vivo in these tumors (Fig. 9) [75]. Currently, everolimus is the only pathway inhibitor available for treating patients with aggressive pituitary tumors and pituitary cancers. To date, there have been reports of seven patients receiving everolimus treatment (comprising three cases of adrenocorticotropic hormone adenomas, one prolactinoma, and three tumors of unspecified subtype). Among these patients, only one exhibited radiographic stability, while the others experienced disease progression [75, 76].

### Immune checkpoint inhibitors

Recent studies have identified lymphocyte infiltration and PD-L1 expression in aggressive pituitary tumors and pituitary cancers [77], suggesting that immune checkpoint inhibitors might offer a new therapeutic approach for these challenging cases (Fig. 6 and Fig. 7). The effectiveness of immune checkpoint inhibitors has been reported in a cohort of seven patients with adrenocorticotropic hormone adenomas and three patients with prolactinomas. The outcomes showed that five patients experienced partial remission as seen on imaging, two patients demonstrated stable disease with no further tumor growth, while three patients continued to exhibit tumor progression [78–81]. There is hope that ongoing clinical trials investigating the combination of nivolumab and ipilimumab (NCT04042753 and NCT02834013) will provide further evidence supporting the efficacy of this treatment strategy for aggressive pituitary tumors and pituitary cancers (Table 5).

**Table 5:** Various targeted therapies for pituitary tumors, summarizing their mechanisms, clinical findings, limitations, emerging strategies, and future directions.

Therapy/Target	Mechanism of action	Clinical findings	Limitations/Challenges	Emerging strategies	Future directions	Ref.
Amino acid metabolic and glucose signaling pathway inhibitors	Target pathways involved in amino acid metabolism and glucose signaling, which may be altered in pituitary tumors, potentially affecting tumor growth and metabolism.	Targeting mTOR has shown some potential in controlling pituitary tumor growth; however, the clinical evidence for efficacy is currently limited and primarily based on preclinical data or indirect evidence from related tumor types.	Limited clinical data on the effectiveness of targeting amino acid metabolic and glucose signaling pathways in pituitary tumors; need for further studies to validate these targets.	Development of inhibitors specifically targeting altered metabolic pathways in pituitary tumors, potentially in combination with existing therapies to enhance treatment response.	Conduct clinical trials to establish the safety and efficacy of targeting metabolic pathways in pituitary tumors; explore the potential for combining these approaches with other targeted therapies for synergistic effects.	[54]
RTKs pathway inhibitors	Target VEGF and its receptors, which are associated with increased vessel density and angiogenesis in aggressive pituitary tumors.	Bevacizumab treatment in 17 patients with aggressive pituitary tumors showed 1 complete remission, 4 partial remissions, 7 stable diseases, and 3 disease progressions. Two patients experienced complications (epistaxis, nephritis).	Limited efficacy with a high rate of stable or progressive disease; potential side effects such as epistaxis and nephritis; variability in response among patients.	Combining VEGF inhibitors like bevacizumab with other agents, such as temozolomide, to enhance efficacy and overcome resistance.	Conduct randomized controlled trials to better understand the effectiveness and safety profile of VEGF inhibitors in combination with other therapies. Explore biomarkers for better patient stratification and prediction of response.	[55, 56]
PI3K/AKT/mTOR pathway inhibitors	Target the PI3K/AKT/mTOR signaling pathway, which is upregulated in anterior pituitary tumors and plays a role in cell growth and survival.	Everolimus, the only available mTOR pathway inhibitor for aggressive pituitary tumors, showed limited effectiveness with 1 patient achieving radiographic stability out of 7 treated; others experienced disease progression.	Poor overall response to everolimus; high rate of disease progression; need for better understanding of pathway dynamics and resistance mechanisms in pituitary tumors.	Exploring combination therapies that target multiple components of the PI3K/AKT/mTOR pathway or combine with other systemic treatments to enhance efficacy.	Further research needed to identify patients most likely to benefit from pathway inhibitors, develop combination regimens, and improve understanding of the molecular drivers of resistance in aggressive pituitary tumors.	[57, 58]
Immune checkpoint inhibitors	Target immune evasion mechanisms through inhibition of PD-1/PD-L1 pathways, potentially enhancing immune-mediated tumor destruction in aggressive pituitary tumors.	In a cohort of 10 patients (7 with adrenocorticotropic hormone adenomas, 3 with prolactinomas), immune checkpoint inhibitors showed 5 partial remissions, 2 stable diseases, and 3 continued tumor progressions.	Variable responses, with some patients continuing to exhibit tumor progression; need for better predictive markers to identify responders; limited long-term outcome data.	Investigating combinations of immune checkpoint inhibitors (e.g., nivolumab + ipilimumab) and other therapies to potentially enhance efficacy and broaden the therapeutic window.	Ongoing clinical trials (NCT04042753, NCT02834013) to establish the efficacy of combination immune checkpoint therapies; further studies to explore the use of biomarkers for better patient selection and to optimize therapeutic combinations.	[59, 63]

## Targeted therapy for schwannomas

Intracranial schwannoma is the most common tumor found in the pontine cerebellum region, with vestibular schwannoma being a histopathologically benign tumor that typically originates from Schwann cells in the eighth cranial nerve, specifically the vestibular nerve [82]. While surgery remains the primary treatment option for vestibular schwannomas, it carries the risk of significant neurological impairment. Therefore, surgical intervention is generally reserved for patients who exhibit symptoms of brainstem compression or have a small but rapidly growing tumor. As research into the signaling pathways involved in vestibular schwannoma growth continues to advance, there is renewed optimism regarding the potential for targeted therapies in the management of this condition.

### RTKs pathway inhibitors

**Fig. 10:**
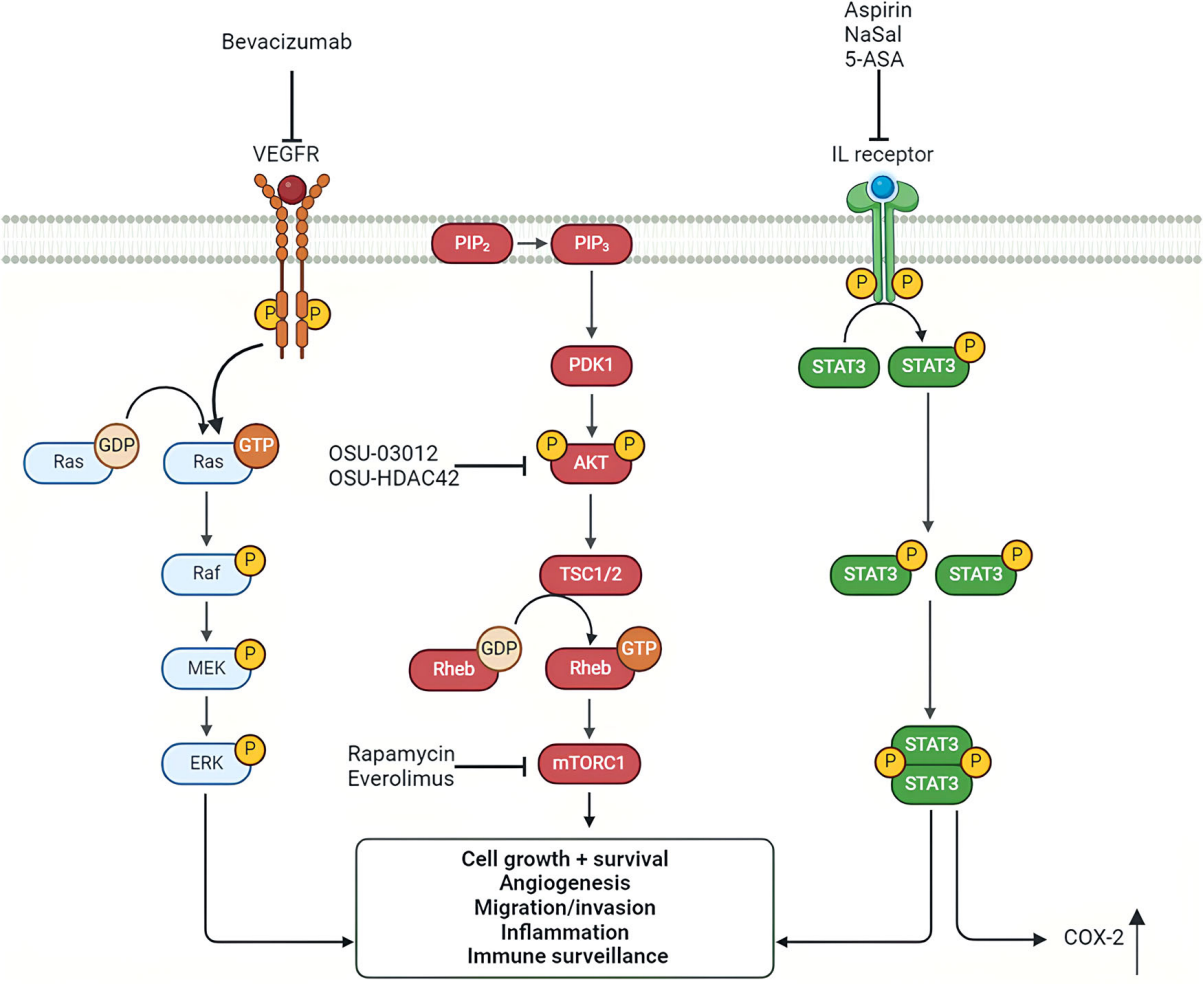
Therapies in pre-clinical and clinical phases to treat schwannomas. The complex interlinked signaling pathways (e.g., VEGF or IL receptor) in the pathogenesis of schwannomas suggest that a combination therapy may provide an ideal therapeutic effect.

EGF has been demonstrated to promote the growth of vestibular schwannomas. Lapatinib, a potent inhibitor of RTKs, has been shown to effectively counteract this growth-promoting effect. A phase II study indicated that lapatinib could reduce tumor volume and improve hearing in patients with progressive vestibular schwannomas [83]. In an immunohistochemical analysis conducted by Huang et al. on 21 vestibular schwannoma specimens associated with neurofibromatosis type 2, VEGF was found to be expressed in 100% of the vestibular schwannomas, and VEGFR-2 was expressed in 32% of the tumor blood vessels [84]. Among the 10 patients who met the study criteria, 9 experienced tumor shrinkage following treatment with the VEGF inhibitor bevacizumab. Specifically, 6 patients exhibited a tumor volume reduction of more than 20% on imaging, and 4 of these patients maintained this reduction without tumor growth during the follow-up period of 11 to 16 months. Additionally, hearing improved in 4 patients, while 2 others maintained stable hearing. These findings suggest that VEGF inhibitors can be effective in reducing tumor volume and improving hearing in some patients with neurofibromatosis type 2 who have vestibular schwannomas (Fig. 10).

### PI3K/AKT and mTOR pathway inhibitors

The mTOR pathway, a downstream signal of the PI3K/AKT pathway, plays a crucial role in integrating signals from various upstream pathways and the local intracellular environment. The membrane protein merlin has been reported to exert a negative regulatory effect on mTORC1, and inhibiting the mTORC1 pathway in tumors lacking merlin could serve as a promising therapeutic target for vestibular schwannomas [85]. Everolimus, a derivative of rapamycin, not only inhibits mTORC1 but also reduces tumor angiogenesis. A study demonstrated that treatment with everolimus significantly reduced the median annual tumor growth rate in patients with neurofibromatosis type 2-associated vestibular schwannomas by 55.6%, decreasing from 67% before treatment to just 0.5% during treatment (Fig. 10) [86].

### Inflammatory factor inhibitors

Several studies have indicated that the immunohistochemical expression of cyclooxygenase-2 (COX-2) is associated with the proliferation of vestibular schwannomas in patients with neurofibromatosis type 2 (Fig. 10) [87]. Prostaglandin E2, catalyzed by COX-2, is involved in various processes such as cell proliferation, apoptosis, angiogenesis, inflammation, and immune surveillance. Therefore, COX-2 inhibitors may hold potential in inhibiting the growth of vestibular schwannomas [88]. Clinical research by Kandathil et al. found a significant negative correlation between aspirin use and vestibular schwannoma growth, suggesting that aspirin may have a potential role in inhibiting the growth of these tumors [89].

## Targeted therapy for craniopharyngioma

Craniopharyngioma is the most common non-neuroepithelial intracranial tumor in minors under 18 years old, accounting for 5% to 11% of intracranial tumors in this age group [90]. Histologically and genomically, craniopharyngiomas are categorized into two distinct types: adamantinomatous craniopharyngiomas (ACP) and papillary craniopharyngiomas (PCP). Despite the potential for a cure through surgical resection and adjuvant radiotherapy, craniopharyngiomas have a high recurrence rate. Research into the molecular mechanisms of craniopharyngioma has identified significant mutations that differentiate ACP from PCP. Specifically, over 90% of ACP cases harbor a CTNNB1 mutation, while more than 90% of PCP cases exhibit a BRAF-V600E mutation (Fig. 10) [91]. Understanding these molecular mechanisms opens new avenues for targeted drug therapy in treating craniopharyngioma.

### RTKs pathway inhibitors

**Fig. 11:**
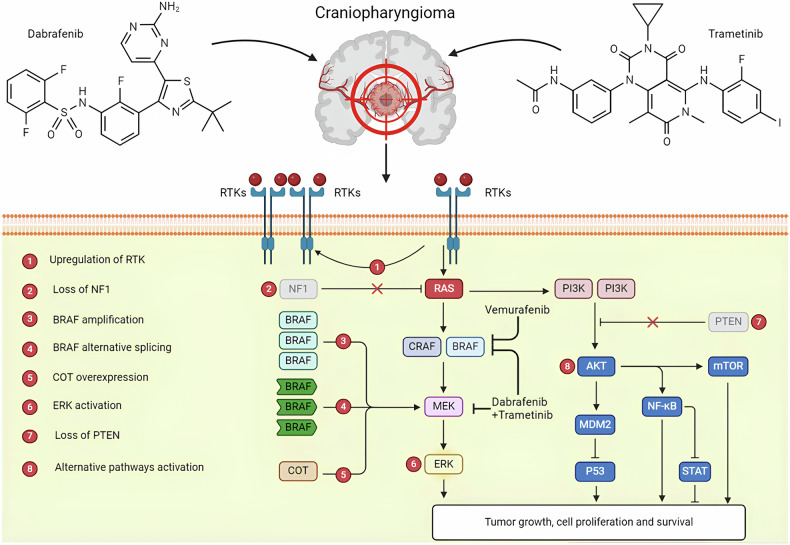
Mechanism of BRAF (BRAF-V600E mutation) positive craniopharyngioma resistance. The oncogenic role of BRAF mutations in papillary craniopharyngiomas is known, where it has been repeatedly proven that combination therapy with dabrafenib and trametinib has a positive effect.

Immunohistochemical analysis has detected the presence of EGFR in most ACP patients, particularly in peripheral nodular cluster cells, suggesting that EGFR signaling may play a role in the cell migration and brain infiltration observed in ACP [92] (Fig. 11). Campanini et al. treated 11 primary ACP cell cultures of human origin with gefitinib, an EGFR signaling pathway inhibitor, and demonstrated that gefitinib could reduce tumor cell motility and myobundle protein expression [92]. This study verified the influence of EGFR signaling on the migration of craniopharyngioma cells in vitro, indicating that EGFR inhibitors may be a promising therapeutic option for ACP.

### BRAF and MEK inhibitors

The BRAF-V600E mutation is highly expressed in PCP, and MEK inhibitors have been found to prevent resistance to BRAF inhibitors during melanoma treatment [22]. Consequently, the combination of BRAF inhibitors and MEK inhibitors is now commonly used to treat tumors with BRAF-V600E mutations (Fig. 11). Brastianos et al. reported a case where a male patient with recurrent craniopharyngioma, harboring a BRAF-V600E mutation, was treated with a combination of the BRAF inhibitor dabrafenib and the MEK inhibitor trametinib [93]. The patient’s tumor volume was reduced by 85% after 35 days of treatment. Additionally, Rostami et al. reported a case of a patient who experienced tumor recurrence five months post-surgery [94]. After 15 weeks of treatment with dabrafenib and trametinib, MRI showed a 91% reduction in tumor volume. There is hope that the ongoing Phase II trial of the BRAF/MEK inhibitors vemurafenib and cobimetinib for the treatment of PCP (NCT03224767) will provide stronger clinical evidence for the efficacy of this combination in BRAF-V600E-positive PCP patients.

### Immune checkpoint inhibitors

Coy et al. utilized circulating immunofluorescence to map the spatial distribution of immune cells within tumor tissues and demonstrated PD-L1 expression in the capsular lining of ACP tumors, as well as PD-1 expression in tumorigenic stem cells [95]. Additionally, PD-L1 expression was found at the stromal-epithelial interface of proliferative tumor cells in PCP. These findings suggest that targeting PD-L1 and/or PD-1 in both craniopharyngioma subtypes could be an effective therapeutic strategy (Figs. 6 and 7).

## The role of the BBB and BTB in brain tumor treatment

Effective treatment of brain tumors such as gliomas, meningiomas, pituitary adenomas, and craniopharyngiomas is often hindered by the presence of the BBB and the blood-tumor barrier BTB. These barriers play crucial roles in maintaining the brain’s homeostasis but also limit the ability of therapeutic agents to reach and effectively treat brain tumors.

The BBB is composed of endothelial cells connected by tight junctions, supported by pericytes and astrocyte end-feet, forming a highly selective barrier that regulates the passage of molecules [96, 97]. This barrier is essential for protecting the brain from toxins and pathogens while maintaining a controlled environment. However, this same selectivity severely restricts the passage of therapeutic drugs, particularly large molecules, posing a challenge in the treatment of aggressive brain tumors like glioblastomas [98]. Unlike the BBB, the BTB that forms around and within brain tumors often exhibits disrupted and irregular vasculature due to rapid and abnormal tumor-induced angiogenesis [99]. This results in regions of varying permeability, which can sometimes allow therapeutic agents to penetrate more effectively than through the intact BBB [100]. However, the heterogeneity of the BTB means that some tumor regions retain BBB-like properties, leading to inconsistent drug distribution [101]. This variability presents a major challenge for achieving uniform treatment coverage in tumors such as gliomas and craniopharyngiomas [102].

High-grade gliomas, including glioblastomas, are particularly difficult to treat due to the intact BBB and inconsistent BTB. The limited penetration of chemotherapeutics and targeted therapies across these barriers results in subtherapeutic concentrations in the tumor [103]. Techniques such as focused ultrasound (FUS) have been used to temporarily disrupt the BBB, enhancing drug delivery and improving treatment outcomes [64].

For meningiomas, which grow outside the brain parenchyma, the BTB can still pose challenges in aggressive or recurrent forms. While surgical resection is typically effective for benign meningiomas, targeted drug delivery for atypical or malignant variants requires methods to enhance BTB permeability [104].

Pituitary adenomas, though often benign, can exhibit invasive growth. The BBB around these tumors can limit systemic therapy, especially for aggressive adenomas resistant to conventional treatments [105]. Drug modification strategies, such as using hormone receptor-targeted therapies, have shown promise in overcoming this barrier [106].

Craniopharyngiomas, located near the pituitary gland and hypothalamus, present unique challenges due to their location and mixed solid-cystic composition. The BTB’s permeability can vary significantly, affecting the consistency of treatment delivery [107]. Localized treatment approaches, such as intracystic injections and nanotechnology-based carriers, are under investigation to bypass these barriers [90].

Innovative approaches are being developed to enhance drug delivery across the BBB and BTB. These include nanoparticle-based delivery - nanoparticles that leverage receptor-mediated transcytosis can facilitate the transport of drugs across the BBB [108]; Focused Ultrasound (FUS) - combined with microbubbles, FUS temporarily disrupts the BBB, allowing drugs to pass through more efficiently [109]; convection-enhanced delivery (CED) - this direct delivery method bypasses the BBB and BTB, ensuring localized drug administration directly into the tumor [110]. Research into combining systemic and localized therapies aims to improve drug penetration and uniform distribution within tumors. Continued exploration of these strategies could enhance treatment outcomes for brain tumors [111].

## Conclusion

Targeted therapy for intracranial tumors represents a rapidly evolving field, driven by advances in molecular biology, genomics, and drug delivery technologies. This review underscores the complexity and diversity of intracranial tumors, including gliomas, meningiomas, pituitary tumors, schwannomas, craniopharyngiomas, ependymomas, medulloblastomas, and primary central nervous system lymphomas. Each tumor type exhibits unique molecular and clinical characteristics, necessitating highly specific therapeutic strategies. The most promising advances have been seen in therapies targeting specific molecular pathways, such as receptor tyrosine kinases (RTKs), PI3K/AKT/mTOR signaling, and RAF/MEK/ERK pathways. For instance, BRAF/MEK inhibitors have shown significant efficacy in treating papillary craniopharyngiomas harboring BRAF-V600E mutations, while VEGF inhibitors like bevacizumab have demonstrated benefits in managing schwannomas and other highly vascularized tumors. Immunotherapies, including immune checkpoint inhibitors and CAR-T cell therapies, offer new avenues for treating otherwise refractory tumors, although their application in brain tumors remains limited by immunosuppressive tumor microenvironments and the BBB. The BBB and BTB continue to be major obstacles, restricting the delivery and effectiveness of systemic therapies. Innovative approaches, such as focused ultrasound, nanoparticle-based delivery systems, and convection-enhanced delivery, are being actively explored to overcome these barriers. Preclinical and early clinical data show potential for these methods to enhance drug penetration, particularly in tumors with intact BBB or heterogeneous BTB characteristics. Despite these advancements, significant gaps remain. Many targeted therapies fail to achieve meaningful survival benefits due to tumor heterogeneity, adaptive resistance mechanisms, and insufficient delivery to the tumor site. For glioblastomas and other high-grade gliomas, where standard therapies provide limited efficacy, the combination of multiple targeted agents or their integration with traditional therapies (e.g., radiotherapy and chemotherapy) may be necessary to overcome resistance and improve outcomes. Similarly, low-grade tumors, while less aggressive, often require prolonged management strategies to prevent recurrence or progression, highlighting the need for durable and safe targeted therapies. Refining molecular profiling techniques to enable precise, patient-specific therapy selection. Investigating synergistic effects of combining targeted therapies with immunotherapies, radiotherapy, or chemotherapy. Developing and optimizing methods to bypass or modulate the BBB and BTB, ensuring uniform drug distribution across heterogeneous tumor regions. Conducting larger, multicenter trials to evaluate the efficacy, safety, and long-term outcomes of emerging targeted therapies, with a focus on clinically meaningful endpoints such as overall survival and quality of life.

In conclusion, while targeted therapies have significantly expanded the treatment landscape for intracranial tumors, their full potential remains untapped. Bridging the gaps in drug delivery, resistance management, and clinical validation will be essential to translating these innovations into tangible benefits for patients. Through sustained research efforts and collaboration across disciplines, the future holds promise for more effective, individualized treatment strategies that improve survival and quality of life for individuals affected by these challenging conditions.
